# Gene regulatory network inference based on a nonhomogeneous dynamic Bayesian network model with an improved Markov Monte Carlo sampling

**DOI:** 10.1186/s12859-023-05381-2

**Published:** 2023-06-24

**Authors:** Jiayao Zhang, Chunling Hu, Qianqian Zhang

**Affiliations:** College of Artificial Intelligence and Big Data, Hefei University, Hefei, 230031 China

**Keywords:** Nonhomogeneous dynamic Bayesian network mode, Multi-change point process, Markov chain Monte Carlo, Gene regulatory network

## Abstract

A nonhomogeneous dynamic Bayesian network model, which combines the dynamic Bayesian network and the multi-change point process, solves the limitations of the dynamic Bayesian network in modeling non-stationary gene expression data to a certain extent. However, certain problems persist, such as the low network reconstruction accuracy and poor model convergence. Therefore, we propose an MD-birth move based on the Manhattan distance of the data points to increase the rationality of the multi-change point process. The underlying concept of the MD-birth move is that the direction of movement of the change point is assumed to have a larger Manhattan distance between the variance and the mean of its left and right data points. Considering the data instability characteristics, we propose a Markov chain Monte Carlo sampling method based on node-dependent particle filtering in addition to the multi-change point process. The candidate parent nodes to be sampled, which are close to the real state, are pushed to the high probability area through the particle filter, and the candidate parent node set to be sampled that is far from the real state is pushed to the low probability area and then sampled. In terms of reconstructing the gene regulatory network, the model proposed in this paper (FC-DBN) has better network reconstruction accuracy and model convergence speed than other corresponding models on the Saccharomyces cerevisiae data and RAF data.

## Introduction

The construction of gene regulatory networks through the analysis of gene expression data is an important method to study gene regulatory relationships, thus aiding in the analysis of biological phenomena [1], for example, studying the etiology of diseases, particularly in developing the target genes at the molecular level of bioinformatics, to better influence the effect of drugs. Given that the gene regulatory networks are frequently constructed from gene expression data, several mathematical models have been introduced and successfully applied in this field, thus providing important computational biology tools for a systematic research on the regulation and patterns of gene transcription in living systems. Representative network models include Boolean network [2, 3], association network [4, 5], differential equation [6–8], and Bayesian network models [9–11]. The Boolean network simplifies the gene state accordingly, and uses Boolean functions instead of differentials and derivatives to describe the relationship between genes. The shortcoming of this model lies in its inaccuracy. Just by using fixed logic rules to describe and reflect the interaction between genes, it cannot accurately describe the real gene regulatory network topology, and it will inevitably cause many problems when discretizing genetic data. The modeling of association network is mainly realized by the degree of association between gene expression data. Mutual information, Pearson correlation coefficient and other measures are usually used to calculate the similarity between genes. If the similarity between gene pairs is higher than a certain threshold, the gene pair is directly connected in the network. The advantage of this method is that the establishment of the model is simple and easy to operate, but there are many false positive edges in the constructed network. Differential equation models can well simulate complex systems, including gene regulatory networks that describe complex regulatory relationships among genes. Although it reflects the internal law, since the establishment of the equation is based on the assumption of the independence of local laws, the deviation is a bit large when making medium and long-term forecasts, and the solution of the differential equation is relatively difficult to obtain.

Recently, the Bayesian network models of gene regulatory networks have been extensively developed owing to their ability to reconstruct directed acyclic graphs, which can describe both the regulatory relationship and the direction of regulation of genes. Friedman et al. have constructed a gene regulatory network containing 800 genes on the basis of the Bayesian network model [12]. However, an unavoidable time delay exists between the regulation of two genes. On the basis of this property, Murphy et al. have proposed a dynamic Bayesian network model to analyze temporal gene expression data [13]. Since real gene networks have cyclic regulatory pathways including feedback loops. When we have time series microarray data, the use of dynamic Bayesian networks (DBNs) is a promising alternative, since DBNs can treat time delay information and can construct cyclic networks. Kim et al. [14] through extensive work, have also improved the dynamic Bayesian network by combining linear or nonlinear models and corresponding biological knowledge.

The structure and parameters of the traditional dynamic Bayesian network model cannot change over time; that is, the time series is required to be a stable distribution generated by a homogeneous Markov chain; thus, the traditional dynamic Bayesian network model is limited by the non-stationary nature of gene expression data. To address this issue, Lèbre et al. [15] have proposed a dynamic Bayesian network model based on a Bayesian regression model (BR-DBN), which incorporates a multi-change point process, thus allowing the network structure and parameters to vary over time. However, the shortcomings of BR-DBN have been exposed in modeling short time-series data of genes. BR-DBN considers dividing data into different segments, and assumes that the regulatory networks in different segments are inconsistent. However, for short time series, even if the environment changes slightly, it is unrealistic for the regulatory network to undergo significant changes. In fact, what changes is only the regulatory strength rather than the regulatory relationship. Such schemes thus lead to overfitting and exaggerated uncertainties for short time series. Subsequently, Dongdelinger et al. [16, 17] have proposed several variants of BR-DBN, on the basis of the assumption that the network structure in different segments is fixed, and only the parameters change. These models all include multi-change point process, but data from different segments must be assigned to different components and do not take into account the temporal information of the data points. To address these problems, the HMM-DBN [18], proposed by Grzegorczyk et al. is based on the assumption of a hidden Markov model dependency structure between time data points. HMM-DBN considers the time order of data points and also does not restrict the distribution of data points. Since the HMM-DBN parameters are node specific, the conditional probabilities of parameters vary among segments. The notable advantage of HMM-DBN is the independence and conjugation of parameters, which can be inferred in a closed form on the basis of the likelihood. Therefore, the inference process has been reduced to sampling the network structure and the polymorphic point process from the posterior distribution through the Markov chain Monte Carlo method.

Herein, to fully exploit the hidden prior information of data points on the basis of HMM-DBN, given the unstable nature of microarray gene expression data, birth action based on the Manhattan distance of data points has been first proposed to improve the rationality of the multi-change point process. Second, according to the sampling network structure of the Markov chain Monte Carlo method, a multi-change point process has been proposed along with the correlations between gene nodes that are calculated in segments, and thus a particle filter is constructed. Pushing nodes to the high probability area causes the sampled particles to be close to the actual state, thereby improving the sampling efficiency, and ultimately the network reconstruction accuracy and the convergence of the model.

This article is divided into four parts. The first part describes the Bayesian regression model combined with the variable point process and the necessary parameter inference. The second part describes the network structure inference combined with particle filters. The third part describes the variable point process. The last section describes the experimental results.

The contributions of this article can be summarized as follows.

1. The dynamic Bayesian network is combined with the multi-variable point process for the analysis of the non-stationarity of gene expression data, including the prior information, variance of the gene data, and Manhattan distance of the mean, for the target gene calculation. The change-point birth process increases the rationality of the multi-change point process.
2. By combining the multi-variable point process, the Pearson correlation coefficient between genes has been calculated segmentally, thus forming a particle filter, which pushes the parent node set close to the true state to the high-probability region and increases the performance of the MCMC sampler.
3. Finally, through experiments using a yeast dataset and nine RAF pathway datasets, the effectiveness, convergence, and model stability of FC-DBN in reconstructing small-scale gene regulatory networks are verified.

## Methods

**Fig. 1:**
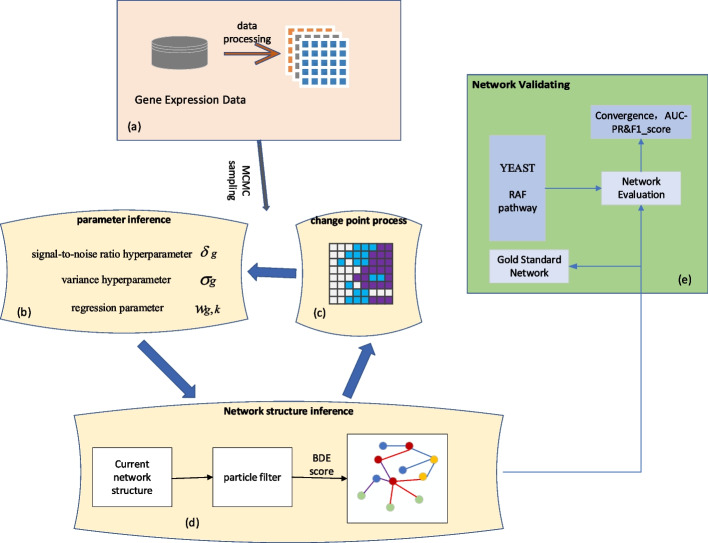
Overall framework of dynamic Bayesian network modeling based on structure prediction: **a** Data are processed into the short time series data required by the model. **b** SNR hyperparameters, regression parameters, and variance parameters are updated through a Markov chain Monte Carlo sampling method. **c** The multi-change point process is updated by the Markov Chain Monte Carlo Sampling method. **d** A particle filter is constructed with a multivariate point process, and the network structure is resampled. **e** Network performance is assessed with standard F-score and AUPR measures, and an experimentally validated biological network

The overall framework of gene regulatory network construction based on a dynamic Bayesian network structure prediction is shown in Fig. 1.

The overall framework of dynamic Bayesian network modeling based on structure prediction is mainly composed of five parts: (a) data preprocessing, (b) Bayesian network parameter learning, (c) multi-change point process, (d) Bayesian network structure learning, and (e) model performance evaluation. Data preprocessing is not described in detail in this paper. “Piecewise Bayesian linear regression” section introduces the parameter inference process of Bayesian network, “Network structure sampling based on node correlation particle filtering” section introduces the structure inference process of Bayesian network, and “Multi-change point process” section introduces the multi-variation point process. “Experiments and results” section presents the performance evaluation.

### Piecewise Bayesian linear regression

The FC-DBN proposed herein is based on piecewise Bayesian linear regression. Its regression equation is:

1 $$y_{g,k} = X_{{\pi_{g,k} }}^{T} w_{g,k} + \varepsilon_{g,k}$$

In each component *k* of FC-DBN, where $g = 1, \ldots ,N$, $N$ is the number of nodes; $y_{g,k}$ is assigned to the observation vector of component *k*, the regression coefficient matrix of the $w_{g,k}$ regression model, $w_{g,k}$ is the set of parent nodes of node *g* in component *k*, $X_{{\pi_{g,k} }}^{T}$ is the observation matrix of the parent node set of node *g* in component *k*, $\varepsilon_{g,k}$ is the noise parameter of the regression model, which obeys a Gaussian distribution with a mean of 0 and a variance of $\sigma_{g}$. Then the regression model likelihood is:

2 $$P\left( {y_{g,k} {|}X_{{\pi_{g,k} }} ,w_{g,k} ,\sigma_{g} } \right) = N(y_{g,k} |X_{{\pi_{g,k} }}^{T} w_{g,k} ,\sigma_{g}^{2} I)$$

**Fig. 2:**
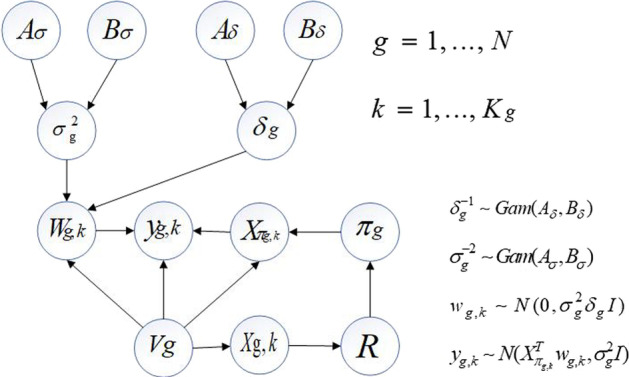
Hierarchy of inhomogeneous dynamic Bayesian network models. The inverse signal-to-noise ratio hyperparameter and the inverse variance hyperparameter are assumed to obey the conjugate gamma distribution, and the regression parameter is assumed to obey the conjugate Gaussian distribution


For the fixed variable point vector $V_{g}$ and the parent node set $\pi_{g}$ of the node, let the regression parameter $w_{g,k}$, the inverse signal-to-noise ratio hyperparameter $\delta_{g}^{ - 1}$, and the inverse variance hyperparameter $\sigma_{g}^{ - 2}$ obey conjugate Gaussian and Gamma distributions. The level-2 hyperparameter $A_{\delta } ,B_{\delta } ,A_{\sigma } ,B_{\sigma }$ is fixed. Figure 2 shows the hierarchical structure of the non-homogeneous dynamic Bayesian network model. The MCMC sampling is according to Eq. (6). Algorithm 1 generates samples from the posterior distribution, and Eq. (3–5) is used to update the hyperparameters.

3 $$\left\{ {\begin{array}{*{20}l} {P\left( {w_{g,k} {|}\sigma_{g}^{2} ,\delta_{g} } \right) = N \left(w_{g,k} |0,\delta_{g} \sigma_{g}^{2} I \right)} \hfill \\ {P \left(w_{g,k} |y_{g,k} ,X_{{\pi_{g,k} }} ,\sigma_{g}^{2} ,\delta_{g} \right) = N((\delta_{g}^{ - 1} I + X_{{\pi_{g,k} }} X_{{\pi_{g,k} }}^{T} )^{ - 1} X_{{\pi_{g,k} }} y_{g,k} ,\sigma_{g}^{2} \left(\delta_{g}^{ - 1} I + X_{{\pi_{g,k} }} X_{{\pi_{g,k} }}^{T} \right)^{ - 1} } \hfill \\ \end{array} } \right.$$

4 $$\left\{ {\begin{array}{*{20}l} {P\left( {\delta_{g}^{ - 1} {|}A_{\delta } ,B_{\delta } } \right) = Gam\left( {\delta_{g}^{ - 1} {|}A_{\delta } ,B_{\delta } } \right) = \frac{{\left[ {B_{\delta } } \right]^{{A_{\delta } }} }}{{\Gamma \left( {A_{\delta } } \right)}}\left[ {\delta_{g}^{ - 1} } \right]^{{A_{\delta } - 1}} e^{{ - B_{\delta } \delta_{g}^{ - 1} }} } \hfill \\ {P(\delta_{g}^{ - 1} |w_{g,k} ,\sigma_{g}^{2} ) = Gam\left( {A_{\delta } + \frac{{K_{g} \left( {\left| {\pi_{g} } \right| + 1} \right)}}{2},B_{\delta } + \frac{1}{{2\sigma_{g}^{2} }}\mathop \sum \limits_{k = 1}^{{K_{g} }} w_{g,k}^{T} w_{g,k} } \right)} \hfill \\ \end{array} } \right.$$

5 $$\left\{ {\begin{array}{*{20}l} {P\left( {\sigma_{g}^{ - 2} {|}A_{\sigma } ,B_{\sigma } } \right) = Gam\left( {\sigma_{g}^{ - 2} {|}A_{\sigma } ,B_{\sigma } } \right) = \frac{{\left[ {B_{\sigma } } \right]^{{A_{\sigma } }} }}{{\Gamma \left( {A_{\sigma } } \right)}}\left[ {\sigma_{g}^{ - 2} } \right]^{{A_{\sigma } - 1}} e^{{ - B_{\sigma } \sigma_{g}^{ - 2} }} } \hfill \\ {P(\delta_{g}^{ - 1} |w_{g,k} ,\sigma_{g}^{2} ) = GamP(\sigma_{g}^{ - 2} |y_{{g,V_{g} }} ,X_{{\pi_{g,} k}} ,\delta_{g} ) = Gam(A_{\sigma } + \frac{T - 1}{2},B_{\sigma } } \hfill \\ { + \frac{{\mathop \sum \nolimits_{k = 1}^{{K_{g} }} \left( {y_{g,k}^{T} \left( {I + \delta_{g} X_{{\pi_{g,k} }}^{T} X_{{\pi_{g,k} }} } \right)^{ - 1} y_{g,k} } \right)}}{2}\left( {A_{\delta } + \frac{{K_{g} \left( {\left| {\pi_{g} } \right| + 1} \right)}}{2},B_{\delta } + \frac{1}{{2\sigma_{g}^{2} }}\mathop \sum \limits_{k = 1}^{{K_{g} }} w_{g,k}^{T} w_{g,k} } \right)} \hfill \\ \end{array} } \right.$$

6 $$P(w_{g,k} ,\delta_{g} ,\sigma_{g}^{2} |D) \propto \mathop \prod \limits_{g} P\left( {\delta_{g} } \right)P\left( {\sigma_{g}^{2} } \right)\mathop \prod \limits_{k} P(w_{g,k} |\delta_{g} ,\sigma_{g} )P(y_{g,k} |X_{{\pi_{g,k} }} ,\sigma_{g} ,w_{g,k} )$$

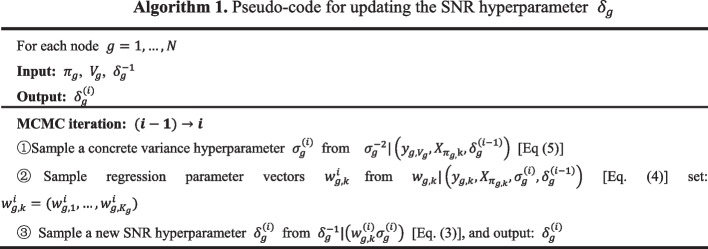


### Network structure sampling based on node correlation particle filtering

**Fig. 3:**
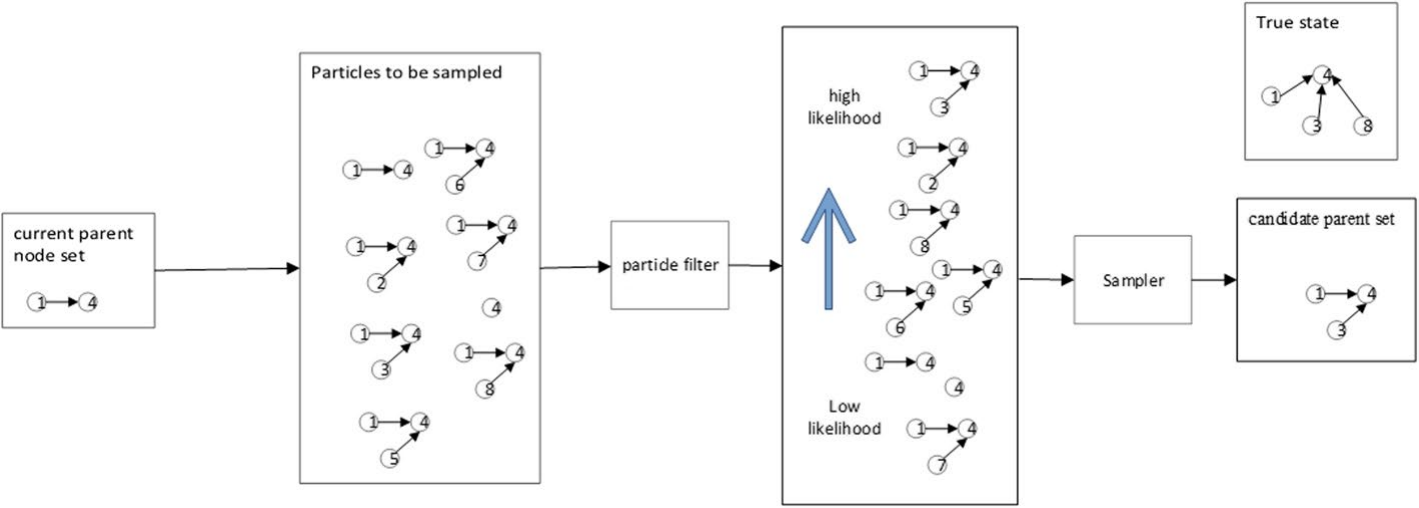
The particle filter is constructed by combining a multi-point process, calculating the Pearson correlation coefficient between nodes in components, and then resampling

The parent node set is ideally sampled close to the actual state. Using MCMC sampling with the parent node set obeying a uniform distribution result in the multiple invalid sampling by the sampler. To overcome this shortcoming, we propose a method to push the parent node set with high similarity to the actual state to the high probability region, and the parent node set dissimilar to the actual state to the low probability region, by using observational information and a variable point process. And the resampling process of the particle filter combined with the multi-variation point process is shown in Fig. 3.

The particle is represented by $\left( {\pi_{g} ,V_{g} ,X_{g,k} } \right)$, $g$ is the node, $\pi_{g}$ is the parent node set, $V_{g}$ is the variable point vector, and $C$ is the auxiliary matrix. At initialization$, \pi_{g} = 0$, $V_{g} = I$, $X_{g,k} = X_{g}$. After one MCMC sampling, the particle state is transferred to the current particle state. According to Algorithm 3, $\pi_{g}^{{\left( {i - 1} \right)}}$ is transformed into $\pi_{g}^{\left( i \right)}$, and according to Algorithm 5, $V_{g}^{{\left( {i - 1} \right)}}$ is transformed into $V_{g}^{\left( i \right)}$.

The candidate parent node set has been obtained by adding or removing parent nodes from the current parent node set. Therefore, we determine whether the parent node set is close to the actual state by constructing a filter matrix based on the correlation between the two nodes. When $g^{\prime} \to g$ is the real state, the node correlation coefficient $R_{{g,g^{\prime}}}$ between nodes $g^{\prime}$ and $g$ is close to 1, and under the action of the filter matrix $R$, the candidate parent node set is expected to be pushed to the high probability region.

The Pearson’s correlation coefficient is used in statistics to measure the linear correlation between two variables [19]. However, the non-stationarity of gene expression data makes analyzing the relationship between gene nodes by Pearson correlation coefficient invalid. We calculate the Pearson’s correlation coefficient between nodes by combining the multi-point process. Through the auxiliary matrix C, the Pearson correlation coefficient of the longer data segment can have a greater effect on the gene node correlation than the shorter date segment. Finally, the particle filter matrix R is obtained.

7 $$R^{i} |\left( {D,V_{g}^{\left( i \right)} ,R^{{\left( {i - 1} \right)}} } \right)\sim R_{{g,g^{\prime}}}^{i} = \left( {R_{{g,g^{\prime}}}^{{\left( {i - 1} \right)}} \times C^{{\left( {i - 1} \right)}} + P_{{X_{g,k} ,X_{{g^{\prime},k^{\prime}}} }} \times \frac{{\left| {X_{g,k} } \right|}}{T}^{{\left( {i - 1} \right)}} } \right)/\left( {C^{i} } \right)$$

where $C^{i} = C^{{\left( {i - 1} \right)}} + \frac{{\left| {X_{g,k} } \right|}}{T}^{{\left( {i - 1} \right)}}$, $|X_{g,k} |$ represents the data length of $|X_{g,k} |$, $k\left( {k = 1, \ldots ,K_{g} } \right)$ is randomly selected with the probability of $\frac{{\left| {X_{g,k} } \right|}}{T}$, $k^{\prime} = V_{{g^{\prime},X_{g,k} }}$. $P_{{X_{g,k} ,X_{{g^{\prime},k^{\prime}}} }}$ is the Pearson’s correlation coefficient, and $P_{{X_{g,k} ,X_{{g^{\prime},k^{\prime}}} }} = \frac{{cov\left( {X_{g,k} ,X_{{g^{\prime}.k^{\prime}}} } \right)}}{{\sigma_{{X_{g,k} }} \sigma_{{X_{{g^{\prime}.k^{\prime}}} }} }}$. Two important properties in the process of building the filter matrix are as follows.

1. In-component data with more data points are relatively easier to use to build filter matrices.
2. In MCMC sampling, the later the sampling, the weaker the update effect of the filter.

On the basis of Algorithm 2, the particles that are close to the real state are pushed to the high probability area.
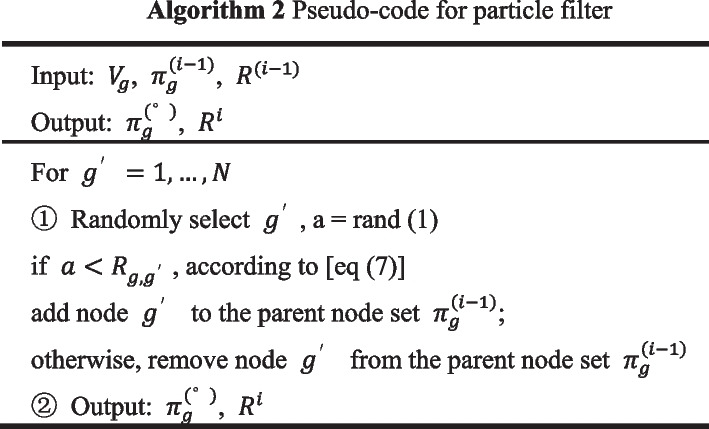


The fixed inverse SNR hyperparameter is $\delta_{g}^{ - 1}$, the regression parameter id $w_{g,k}$, the inverse variance hyperparameter is $\sigma_{g}^{ - 2}$, and the variable point component vector is $V_{g}$. Let the network structure $M = \left( {\pi_{1} , \ldots ,\pi_{N} } \right)$; then the probability distribution of the network structure is:

8 $$P\left( M \right) = \mathop \prod \limits_{g = 1}^{N} P\left( {\pi_{g} } \right)$$

For each node g, the conditional probability of its parent node set $\pi_{g}$ is:

9 $$P(\pi_{g} |D,V_{g} ,\delta_{g} ) \propto P(y_{{g,V_{g} }} |X_{{\pi_{g} ,k}} ,\delta_{g} )$$

According to the Metropolis–Hastings sampling (M–H sampling) criterion, the probability that the candidate parent node sets $\pi_{g}^{\left(^\circ \right)}$ is accepted is:

10 $$A\left( {\pi_{g}^{{\left( {i - 1} \right)}} \to \pi_{g}^{\left(^\circ \right)} } \right) = min\left( {1,\frac{{P(y_{{g,V_{g} }} |X_{{\pi_{g}^{\left(^\circ \right)} ,k}} ,\delta_{g} )}}{{P(y_{{g,V_{g} }} |X_{{\pi_{g}^{{\left( {i - 1} \right)}} ,k}} ,\delta_{g} )}} \times \frac{{P\left( {\pi_{g}^{\left(^\circ \right)} } \right)}}{{P\left( {\pi_{g}^{{\left( {i - 1} \right)}} } \right)}} \times \frac{{\left| {S\left( {\pi_{g}^{{\left( {i - 1} \right)}} } \right)} \right|}}{{\left| {S\left( {\pi_{g}^{\left(^\circ \right)} } \right)} \right|}}} \right)$$

If the action is accepted, then: $\pi_{g}^{\left( i \right)} = \pi_{g}^{\left(^\circ \right)}$; otherwise, $\pi_{g}^{\left( i \right)} = \pi_{g}^{{\left( {i - 1} \right)}}$.
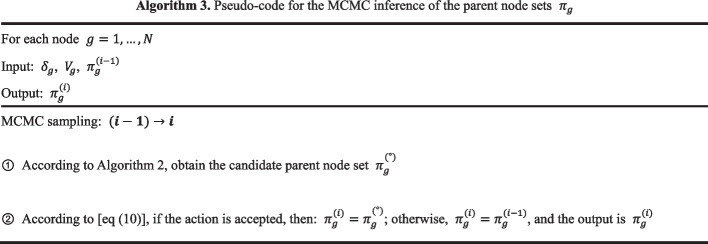


### Multi-change point process

**Fig. 4:**
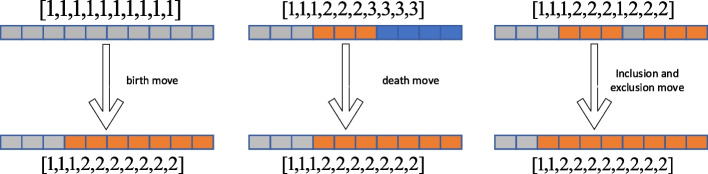
Three move schemes for the multi-change process: birth move, death move, inclusion move, and exclusion move


The above reasoning is based on the assumption of that the component vector $V_{g}$ is fixed. This section describes the sampling process of the component vector $V_{g}$. The component vector changes are determined by the moves of birth, death, and complementary inclusion of the transition point. Figure 4 is a schematic diagram of three actions.

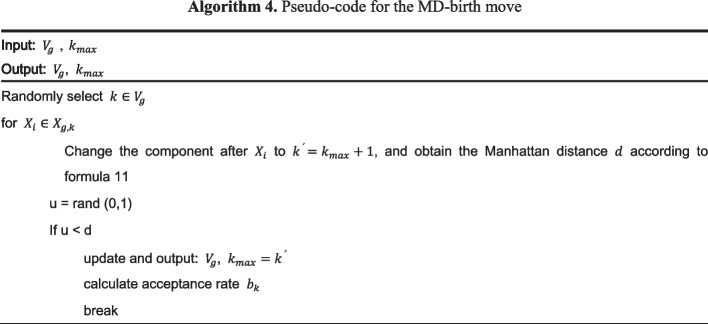


We propose a birth move based on the Manhattan distance of data points, and assume that the mean and variance of observation vectors of different components will differ. According to this assumption, by calculating the Manhattan distance of the mean and variance within different components, the birth move will tend to move in the direction of the larger Manhattan distance

11 $$d = \lambda \left( {\left| {var\left( {X_{g,k} } \right) - var\left( {X_{{g,k^{\prime}}} } \right)\left| + \right|u\left( {X_{g,k} } \right) - u\left( {X_{{g,k^{\prime}}} } \right)} \right|} \right)$$

where ${b}_{k}$, ${d}_{k}$, and ${r}_{k}$ represent the acceptance rates of the birth move, death move, and inclusion and exclusion move actions, respectively, which can be obtained according to the method proposed by Grzegorczyk et al. The RJ-MCMC algorithm steps for updating the changepoint are shown in Algorithm 5.


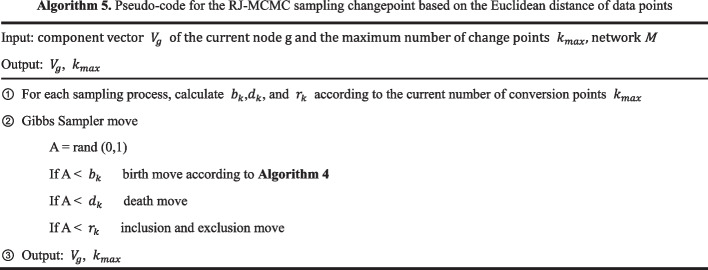


The algorithm flow of the FC-DBN is shown in Algorithm 6.
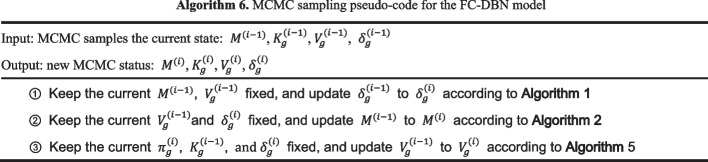


## Experiments and results

### Experimental settings

**Fig. 5:**
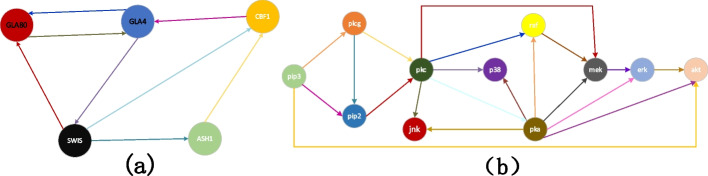
**a** The gold standard network of the yeast data. **b** The gold standard network of the RAF pathway data

The experimental section is divided into three parts using a yeast dataset and nine datasets of the RAF pathway to evaluate the FC-DBN network reconstruction accuracy, model stability, and convergence of MCMC sampling. The yeast dataset containing five gene nodes is a small network structure designed by Cantone et al. The authors measured the expression levels of these genes in vivo through real-time quantitative polymerase chain reaction over 37 time points. Cantone et al. have changed the carbon source from galactose to glucose during the experiment. The dataset contains 16 measurements in galactose and 21 measurements in glucose; the observed value of g at each node was recorded. Owing to the error in washing while changing glycogen, the two first measurement values have been removed to obtain a 5 × 35 dataset [4]. The RAF pathway data with 11 nodes has been provided by Grzegorczyk et al. [18]. The RAF pathway shows the regulatory interactions among the following *n* = 11 proteins: PIP3, PLCG, PIP2, PKC, PKA, JNK, P38, RAF, MEK, ERK, and AKT. There are 20 regulatory interactions (directed edges) in the RAF pathway. Figure 5 shows the yeast network structure and the topology of the RAF pathway.

According to the posterior probability ${e}_{n,j}\in (\mathrm{0,1})$ of the existence of each edge, $E(\xi )$ is defined as the set of all edges whose posterior probability exceeds a threshold ξ, where $\xi \in [\mathrm{0,1}]$. According to $E(\xi )$, the numbers of true positive $TP[\xi ]$, false positive $FP[\xi ]$, and false negative $FN[\xi ]$ are determined. The network reconstruction ability of the model is evaluated with two evaluation metrics.

Equations 12–14 show the evaluation index expression. The precision-recall (PR) curve is obtained by connecting adjacent points through nonlinear interpolation. The area under the PR curve (AUC-PR) is a quantitative measure that can be obtained by numerically integrating the PR curve [21]. The larger the AUC-PR and ${F}_{score}$ value, the stronger the network reconstruction ability of the model.

12 $$R\left[ \xi \right] = TP\left[ \xi \right]/\left( {TP\left[ \xi \right] + FN\left[ \xi \right]} \right)$$

13 $${\text{P}}\left[ {\upxi } \right] = {\text{TP}}\left[ {\upxi } \right]/\left( {{\text{TP}}\left[ {\upxi } \right] + {\text{FP}}\left[ {\upxi } \right]} \right)$$

14 $${\text{F}}_{{{\text{score}}}} = \left( {2 \times {\text{R}}\left[ {\upxi } \right] \times {\text{P}}\left[ {\upxi } \right]} \right)/\left( {{\text{R}}\left[ {\upxi } \right] + P\left[ \xi \right]} \right)$$

To assess convergence, we consider scatter plots of the edge scores of ten independent MCMC simulations on the same dataset. We assume that the current number of MCMC simulations is $I$, the burning rate is burn_in, and ${net(n,j)}^{i}=1$ indicates that edge $n\to j$ exists when the number of iterations is $i$; otherwise, ${net(n,j)}^{i}=0$. We perform Q independent replicates of MCMC sampling. Plots of a scatterplot with ${average\_edge\_scores}_{(n,j)}$ values as the vertical axis and ${edge\_scores}_{(n,j)}$ values as the horizontal axis are constructed.

15 $$edge\_scores_{{\left( {n,j} \right)}}^{q} = \frac{{\mathop \sum \nolimits_{i = burn\_in + 1}^{I} { }net\left( {n,j} \right)^{i} }}{I - burn\_in}$$

16 $$average\_edge\_scores_{{\left( {n,j} \right)}} = \frac{{\mathop \sum \nolimits_{q = 1}^{Q} edge\_scores_{{\left( {n,j} \right)}}^{q} }}{Q}$$

### Experimental results

#### Network reconstruction accuracy evaluation

A particle filter is constructed to improve the efficiency of the MCMC sampler. Table 1 shows the experimental results of the ratio of acceptance times to sampling times for the MCMC sampling network structure. The MCMC sampler of FC-DBN performs significantly better than HMM-DBN. The efficiency of HMM-DBN’s MCMC sampler is less than 40% on the yeast dataset and less than 50% even on the RAF pathway data. Therefore, more than half the sampler's performance is wasted. However, compared with that of HMM-DBN, the performance of FC-DBN's MCMC sampler is greatly improved, since we constructed a particle filter to cause the particles to be sampled closer to the actual state. The improvement in the performance of the MCMC sampler enables higher network reconstruction accuracy to be obtained with fewer MCMC samples.

**Table 1:** Comparison of acceptance rates of HMM-DBN and FC-DBN samplers

DATA	HMM-DBN			FC-DBN		
	Accept times	MCMC times	Ratio (%)	Accept times	MCMC times	Ratio (%)
*YEAST*						
	7617	23,893	31	18,243	25,115	72
data_1	21,065	54,263	39	42,579	54,952	77
data_2	23,911	53,135	45	42,324	55,136	76
data_3	22,310	53,058	42	40,971	55,092	74
data_4	24,228	54,223	44	42,072	54,685	76
*RAF*						
data_5	21,144	54,716	38	42,056	54,785	76
data_6	24,638	54,719	45	43,004	54,766	78
data_7	22,518	54,226	41	39,359	54,976	71
data_8	21,666	55,056	39	43,062	55,425	77
data_9	22,452	54,937	40	40,260	55,513	72

**Fig. 6:**
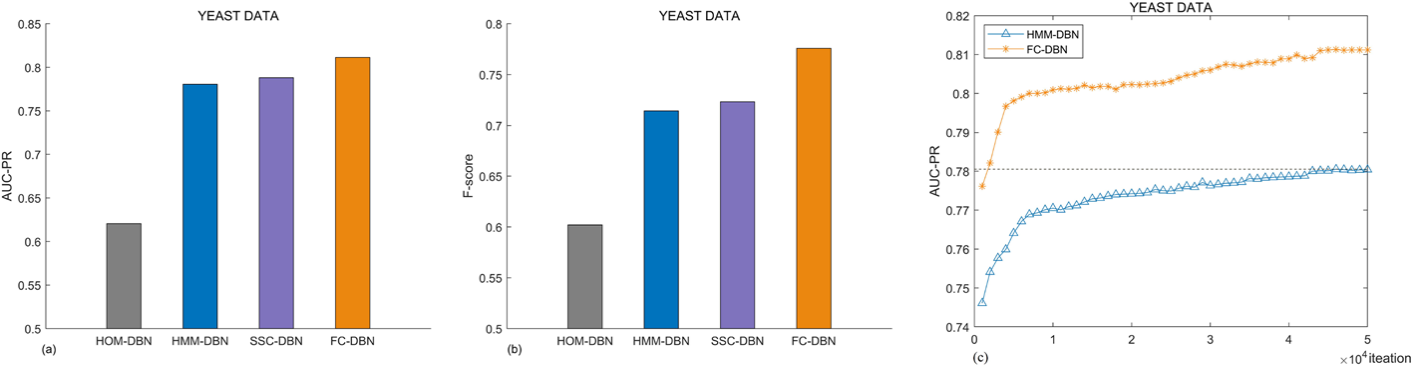
Comparison of network reconstruction capabilities of different models under different evaluation indicators: **a** evaluation of network reconstruction ability with the AUC-PR evaluation index. **b** Evaluation of network reconstruction ability with the F-score evaluation index. **c** Comparison of network reconstruction capability of HMM-DBN and FC-DBN under different MCMC sampling times

**Fig. 7:**
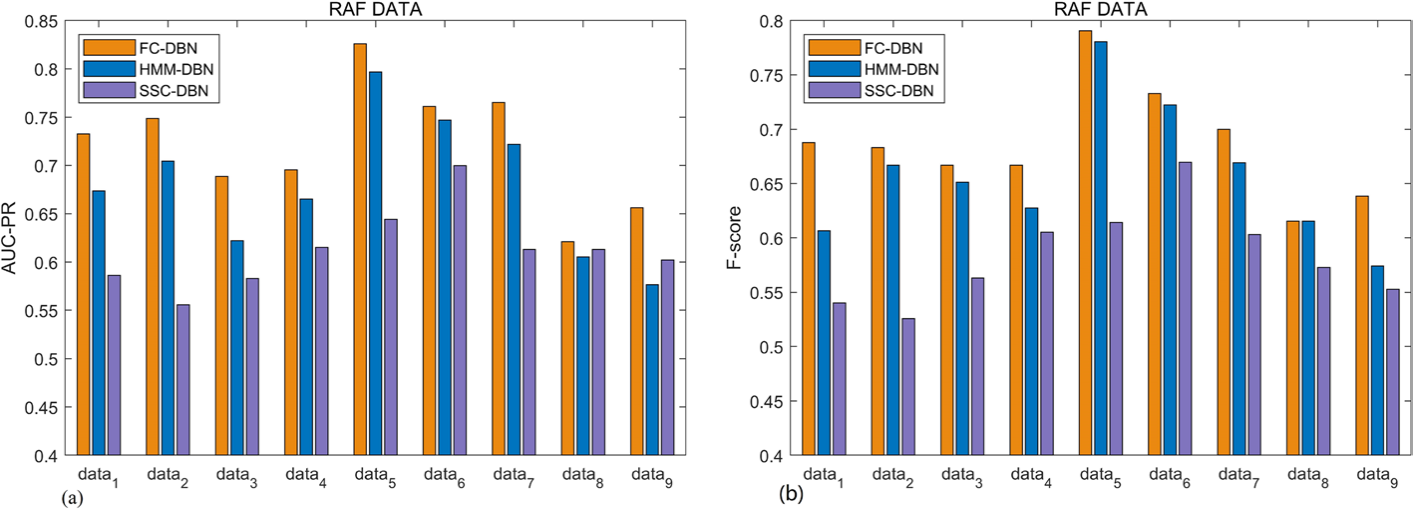
AUC-PR and F-score evaluations of three different models on nine sets of RAF data: **a** evaluation of network reconstruction ability with the AUC-PR evaluation index. **b** Evaluation of network reconstruction ability with the F-score evaluation index

We have used 50 independent MCMC samples to obtain 50 sets of AUC-PR and F-scores, with the mean as the final criterion. Figure 6a shows the AUC-PR of different models under yeast data, and Fig. 6b shows the F-score of different models under yeast data, where HOM-DBN is a dynamic Bayesian network model that does not include a multivariate point process. The network reconstruction accuracy of the dynamic Bayesian network model (HMM-DBN, FC-DBN) combined with the multi-change point process performs significantly better than that of HOM-DBN. Owing to the improved performance of the MCMC sampler, the AUC-PR and F-score values of the FC-DBN network have improved by 3% and 5%, respectively, with respect to those of the HMM-DBN. Figure 6c shows the yeast network reconstruction accuracy at different MCMC sampling times. Although the FC-DBN model does not converge at 1500 MCMC samples, the same average network reconstruction accuracy as that of HMM-DBN can be obtained with 50,000 MCMC samples. Figure 7a shows the comparison of AUC-PR values under three different models: SSC-DBN [20], HMM-DBN, and FC-DBN. Figure 7b shows the comparison of F-scores of the three models. Tables 2 and 3 give the specific values.

**Table 2:** AUC-PR estimates of three models on nine sets of RAF data

Model	RAF data								
	data1	data2	data3	data4	data5	data6	data7	data8	data9
SSC-DBN	0.586	0.556	0.583	0.615	0.644	0.700	0.613	0.613	0.602
HMM-DBN	0.674	0.704	0.622	0.665	0.797	0.747	0.722	0.605	0.577
FC-DBN	0.732	0.749	0.689	0.695	0.826	0.761	0.765	0.621	0.656

**Table 3:** F-score estimates of three models on nine sets of RAF data

Model	RAF data								
	data1	data2	data3	data4	data5	data6	data7	data8	data9
SSC-DBN	0.540	0.526	0.563	0.605	0.614	0.670	0.603	0.573	0.553
HMM-DBN	0.607	0.667	0.651	0.628	0.781	0.722	0.670	0.615	0.574
FC-DBN	0.688	0.683	0.667	0.667	0.791	0.733	0.700	0.615	0.638

We have used 50 independent MCMC samples to obtain 50 sets of AUC-PR and F-scores, with the mean as the final criterion. Figure 6a shows the AUC-PR of different models under yeast data, and Fig. 6b shows the F-score of different models under yeast data, where HOM-DBN is a dynamic Bayesian network model that does not include a multivariate point process. The network reconstruction accuracy of the dynamic Bayesian network model (HMM-DBN, FC-DBN) combined with the multi-change point process performs significantly better than that of HOM-DBN. Owing to the improved performance of the MCMC sampler, the AUC-PR and F-score values of the FC-DBN network have improved by 3% and 5%, respectively, with respect to those of the HMM-DBN. Figure 6c shows the yeast network reconstruction accuracy at different MCMC sampling times. Although the FC-DBN model does not converge at 1500 MCMC samples, the same average network reconstruction accuracy as that of HMM-DBN can be obtained with 50,000 MCMC samples. Figure 7a shows the comparison of AUC-PR values under three different models: SSC-DBN [20], HMM-DBN, and FC-DBN. Figure 7b shows the comparison of F-scores of the three models. Tables 2 and 3 give the specific values.

From Figs. 6 and 7, we can find that in the RAF pathway data data5, data6 and data8, the network reconstruction accuracy of SSC-DBN compared with HMM-DBN does not have a more obvious improvement than that of YEAST data. After analyzing the main differences in data characteristics and models, there may be two reasons:

1. RAF data has obvious segmentation characteristics. Compared with SSC-DBN, HMM-DBN, which performs data segmentation based on hidden Markov model To a certain extent, it makes up for the SSC-DBN with sequential coupling parameters.
2. The coupling relationship between the segments of RAF data is not strong enough. When the data segmentation is not particularly in line with the actual situation, the coupling parameters cannot fully compensate for the segmentation The impact of the segment.

#### Model convergence evaluation

**Fig. 8:**
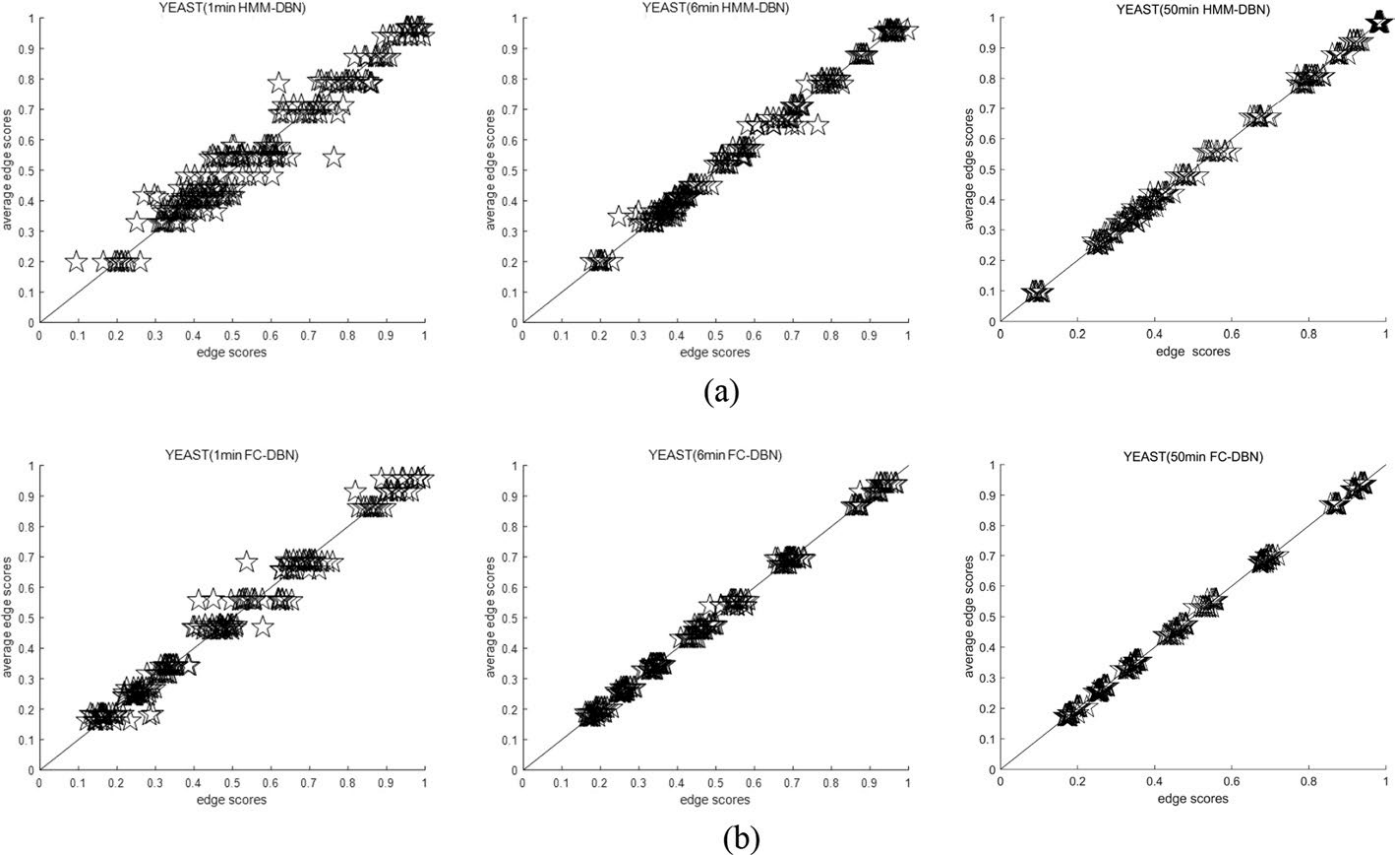
Convergence effect of HMM-DBN and FC-DBN under different MCMC simulation time: **a** convergence effect of HMM-DBN under MCMC simulation for 1 min, 6 min, and 50 min. **b** Convergence effect of FC-DBN under MCMC simulation for 1 min, 6 min, and 50 min

**Fig. 9:**
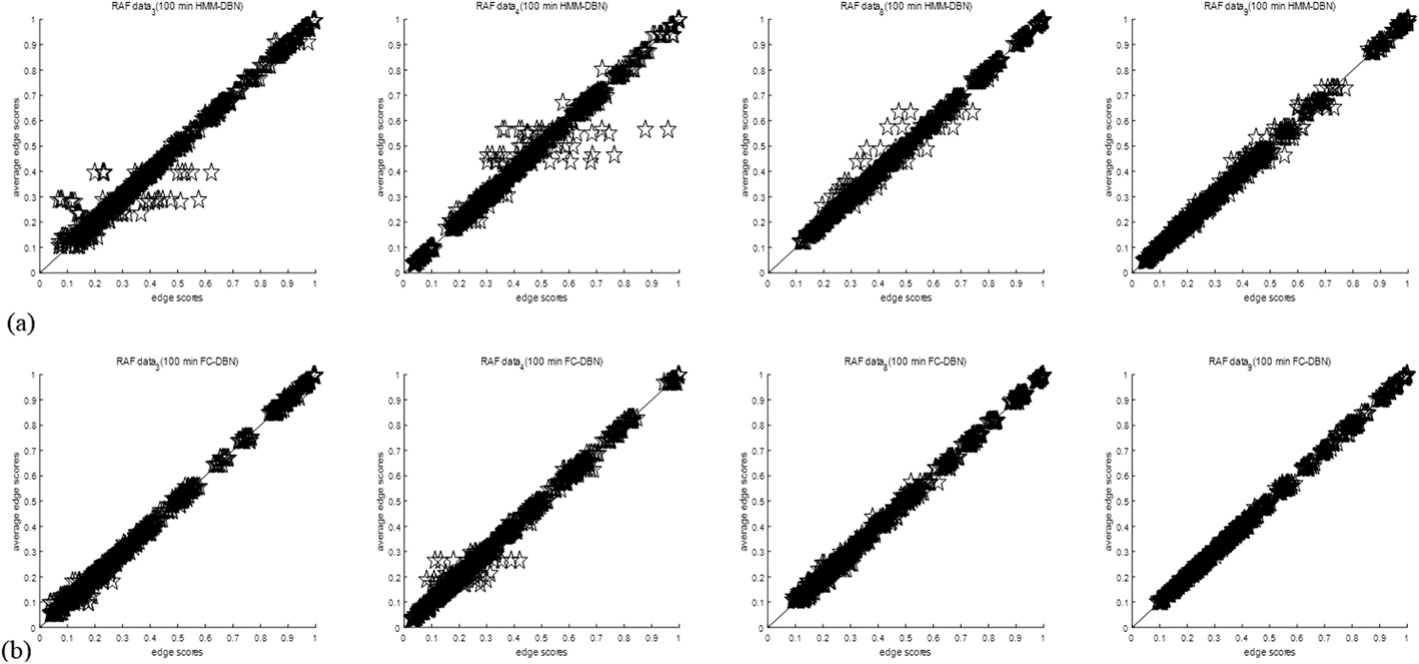
Convergence scatter plot of HMM-DBN and FC-DBN at an MCMC simulation time of 100 min on four sets of RAF data: **a** convergence scatter plot of HMM-DBN for four groups of RAF data. **b** Convergence scatter plot of FC-DBN for four groups of RAF data

The simulation platform had the following specifications. ① Processor: Intel Core i5-9500, CPU 3.0 GHz. ② Installed memory (RAM): 8 GB. ③ Hard disk: 1 TB. ④ Software: MATLAB R2018b. On the yeast data, we performed MCMC simulations at three different times. The MCMC simulation for each time consisted of ten independent MCMC simulations. The edge score and the average edge score have been calculated, and a scatter plot was drawn. Figures 8 and 9 show the MCMC simulation convergence of FC-DBN and HMM-DBN under different conditions. Under the same conditions, the closer edge score of scatter plot to y = x, results in better convergence effect.

Supplementary experiments were performed here and modified in the manuscript. The variance of each edge is obtained from 10 independent MCMC samples, and the variance of all edges is summed. We believe that the smaller the sum of the variances, the better the model convergence. Table 4 shows the comparison of the variance of edge scores between HMM-DBN and FC-DBN under different time losses. Obviously, FC-DBN has a smaller variance than HMM-DBN edge scores. Concomitantly, with respect to the MCMC simulation time, the scatter plot of FC-DBN is closer to the $y=x$ line than that of HMM-DBN. Therefore, the convergence of FC-DBN is better than that of HMM-DBN for the yeast data.

**Table 4:** Comparison of variance of marginal scores under different models and different time losses in yeast data

Model/time	1 min	6 min	50 min
HMM-DBN	$4.7 \times 10^{ - 3}$	$9.1 \times 10^{ - 3}$	$7.1 \times 10^{ - 4}$
FC-DBN	$3.0 \times 10^{ - 3}$	$4.4 \times 10^{ - 3}$	$5.5 \times 10^{ - 3}$

**Fig. 10:**
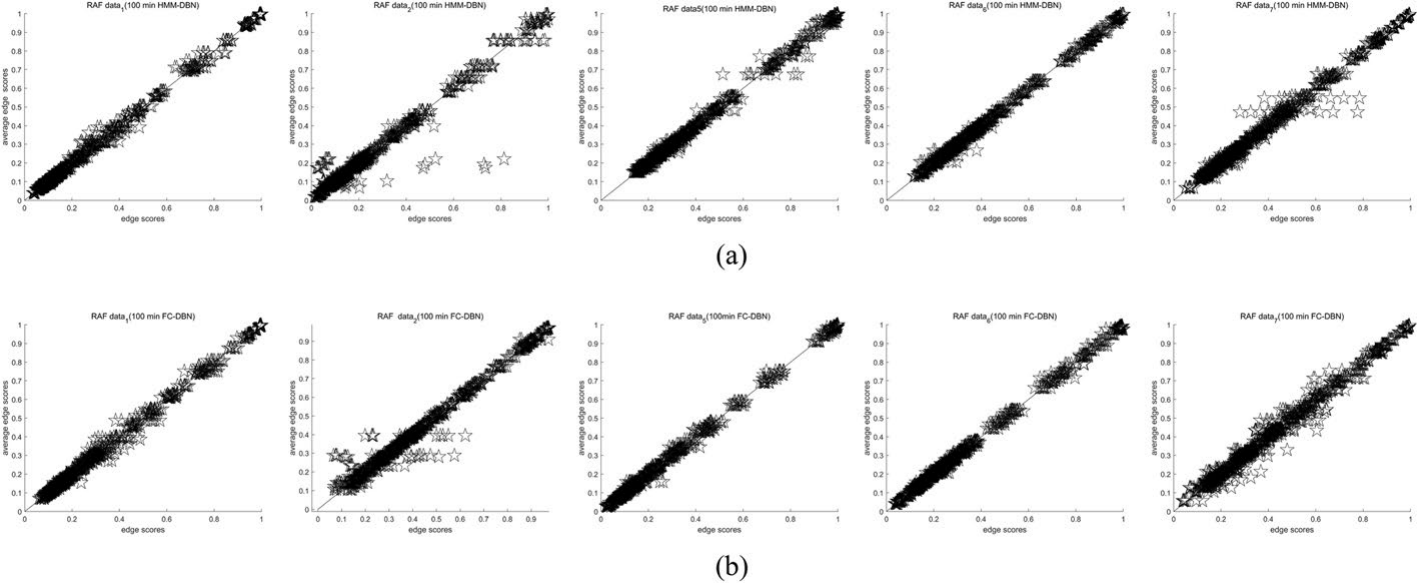
Convergence scatter plot of HMM-DBN and FC-DBN at an MCMC simulation time of 100 min on five sets of RAF data: **a** convergence scatter plot of HMM-DBN for five groups of RAF data. **b** Convergence scatter plot of FC-DBN for four groups of RAF data

Table 5 shows the comparison of HMM-DBN and FC-DBN loss lower edge score variance with a time loss of 100 min. Obviously, the variance of FC-DBN is smaller than the edge score of HMM-DBN. Among them, under four sets of data (3, 4, 8, 9) FC-DBN has a significant improvement in convergence performance compared to HMM-DBN. The scattergram in Fig. 9b is closer to the y = x line than the scattergram in Fig. 9a. Although Fig. 10 shows the scatterplots under the other five sets of data, the convergence of FC-DBN is not significantly better than that of HMM-DBN. But from the variance comparison of edge scores in Table 5, it can be seen that the convergence performance of FC-DBN is still slightly better than that of HMM-DBN.

**Table 5:** Comparison of marginal score variance of different models under 9 sets of data in RAF pathway

Model/data	Data1	Data2	Data3	Data4	Data5	Data6	Data7	Data8	Data9
HMM-DBN	$4.3 \times 10^{ - 2}$	$6.4 \times 10^{ - 2}$	$3.8 \times 10^{ - 2}$	$4.6 \times 10^{ - 2}$	$4.8 \times 10^{ - 2}$	$3.6 \times 10^{ - 2}$	$6.3 \times 10^{ - 2}$	$3.3 \times 10^{ - 2}$	$2.9 \times 10^{ - 2}$
FC-DBN	$3.6 \times 10^{ - 2}$	$5.5 \times 10^{ - 2}$	$2.3 \times 10^{ - 2}$	$3.3 \times 10^{ - 2}$	$3.9 \times 10^{ - 2}$	$3.5 \times 10^{ - 2}$	$5.9 \times 10^{ - 2}$	$1.9 \times 10^{ - 2}$	$1.5 \times 10^{ - 2}$

## Conclusion 

FC-DBN has been proposed owing to the low efficiency of MCMC samplers during the DBN network reconstruction. The purpose of FC-DBN is to provide a sampling space proximate to the real state space for the network structure sampling of DBN through the particle filter step, which must push TP edges and TN edges to high-probability regions and low-probability regions. Therefore, in the network structure sampling stage, the efficiency of the MCMC sampler is greatly improved. Furthermore, combining the birth action of Manhattan distance makes the multi-change point process more reasonable, thus establishing the basis for building particle filters.

In our experiments, we have first evaluated the FC-DBN and HMM-DBN MCMC samplers and found that FC-DBN resulted in a significantly higher sampler efficiency than HMM-DBN. Then, we have compared the accuracy of network reconstruction, for the yeast data, for the dynamic Bayesian network model (HOM-DBN) without the combination of the multi-point process, the dynamic Bayesian network model (HMM-DBN) combined with the multi-point process, and the combination of the multi-point process and the dynamic Bayesian network model of particle filter (FC-DBN). Experimental comparisons have indicated that HMM-DBN has better network reconstruction ability than HOM-DBN. With the improved MCMC sampler, FC-DBN can obtain the same network reconstruction accuracy as HMM-DBN with shorter sampling times, while improving the network reconstruction ability. Since FC-DBN adds a particle filter step, which inevitably increases the time loss, the result comparisons have been considered only for the same times in the convergence analysis with HMM-DBN. Through the experimental comparison of the yeast data and the nine sets of data of the RAF pathway, we have found that FC-DBN has a better convergence than HMM-DBN. This convergence owes to the sampling progress of MCMC that leads to the convergence of the multi-point process, and hence the particle filter can push the MCMC sampling space.

However, the model proposed in this paper also has some problems. First, especially in the face of a large multi-node network structure, the time overhead of the algorithm increases exponentially; second, in the face of some specific data sets, satisfactory results cannot be obtained.

## Data Availability

The datasets analysed during the current study are available in the figshare repository, https://figshare.com/s/96f578777aa6b43f3638.

## References

[1] Emmert-Streib F, Dehmer M, Haibe-Kains B (2014). Gene regulatory networks and their applications: understanding biological and medical problems in terms of networks. Front Cell Dev Biol.

[2] Shmulevich I, Dougherty ER, Kim S (2002). Probabilistic Boolean networks: a rule-based uncertainty model for gene regulatory networks. Bioinformatics.

[3] Timmermann T, González B, Ruz GA (2020). Reconstruction of a gene regulatory network of the induced systemic resistance defense response in Arabidopsis using Boolean networks. BMC Bioinform.

[4] Cantone I, Marucci L, Iorio F (2009). A yeast synthetic network for in vivo assessment of reverse-engineering and modeling approaches. Cell.

[5] Pirgazi J, Khanteymoori AR (2018). A robust gene regulatory network inference method base on Kalman filter and linear regression. PLoS ONE.

[6] Chen S, Shojaie A, Witten DM (2017). Network reconstruction from high-dimensional ordinary differential equations. J Am Stat Assoc.

[7] Deng Y, Zenil H, Tegnér J (2017). HiDi: an efficient reverse engineering schema for large-scale dynamic regulatory network reconstruction using adaptive differentiation. Bioinformatics.

[8] Ma B, Fang M, Jiao X (2020). Inference of gene regulatory networks based on nonlinear ordinary differential equations. Bioinformatics.

[9] Li Z, Li P, Krishnan A (2011). Large-scale dynamic gene regulatory network inference combining differential equation models with local dynamic Bayesian network analysis. Bioinformatics.

[10] Michailidis G, d’Alché-Buc F (2013). Autoregressive models for gene regulatory network inference: sparsity, stability and causality issues. Math Biosci.

[11] Buetti-Dinh A, Herold M, Christel S (2020). Reverse engineering directed gene regulatory networks from transcriptomics and proteomics data of biomining bacterial communities with approximate Bayesian computation and steady-state signalling simulations. BMC Bioinform.

[12] Friedman N (2004). Inferring cellular networks using probabilistic graphical models. Science.

[13] Murphy K, Mian S. Modelling gene expression data using dynamic Bayesian networks. Technical report, Computer Science Division, University of California, Berkeley, CA; 1999.

[14] Kim SY, Imoto S, Miyano S (2003). Inferring gene networks from time series microarray data using dynamic Bayesian networks. Brief Bioinform.

[15] Lèbre S, Becq J, Devaux F, Stumpf MP, Lelandais G (2010). Statistical inference of the time-varying structure of gene-regulation networks. BMC Syst Biol.

[16] Dondelinger F, Lebre S, Husmeier D, Furnkranz J, Joachims T (2010). Heterogeneous continuous dynamic Bayesian networks with flexible structure and inter-time segment information sharing. International conference on machine learning (ICML).

[17] Dondelinger F, Lèbre S, Husmeier D (2013). Non-homogeneous dynamic Bayesian networks with Bayesian regularization for inferring gene regulatory networks with gradually time-varying structure. Mach Learn.

[18] Grzegorczyk M (2016). A non-homogeneous dynamic Bayesian network with a hidden Markov model dependency structure among the temporal data points. Mach Learn.

[19] Cohen I, Juang Y, Chen J, Benesty J. Pearson correlation coefficient. In: Noise reduction in speech processing. Springer, Berlin/Heidelberg, Germany, pp. 1–4; 2009.

[20] ShafieeKamalabad M, Grzegorczyk M (2018). Improving nonhomogeneous dynamic Bayesian networks with sequentially coupled parameters. Stat Neerl.

[21] Davis J, Goadrich M. The relationship between precision-recall and roc curves. In: Proceedings of the 23rd international conference on machine learning, New York, NY, USA, 25–29 June 2006.

